# Differential iNKT and T Cells Activation in Non-Alcoholic Fatty Liver Disease and Drug-Induced Liver Injury

**DOI:** 10.3390/biomedicines10010055

**Published:** 2021-12-28

**Authors:** Estefanía Caballano-Infantes, Alberto García-García, Carlos Lopez-Gomez, Alejandro Cueto, Mercedes Robles-Diaz, Aida Ortega-Alonso, Flores Martín-Reyes, Ismael Alvarez-Alvarez, Isabel Arranz-Salas, Francisco Ruiz-Cabello, Isabel M. Lucena, Eduardo García-Fuentes, Raúl J. Andrade, Miren García-Cortes

**Affiliations:** 1 Departamento de Farmacología, Facultad de Medicina, Universidad de Málaga, 29010 Málaga, Spain; estcabinf@gmail.com (E.C.-I.); acuetos@outlook.es (A.C.); 2 UGC de Aparato Digestivo, Hospital Universitario Virgen de la Victoria, Universidad de Málaga, 29010 Málaga, Spain; albergarcias@gmail.com (A.G.-G.); carlos.lopez@ibima.eu (C.L.-G.); mrobles@uma.es (M.R.-D.); aida_ortega_alonso@hotmail.com (A.O.-A.); floresmarey@hotmail.com (F.M.-R.); iaalvarez@uma.es (I.A.-A.); andrade@uma.es (R.J.A.); mirengar1@hotmail.com (M.G.-C.); 3 Instituto de Investigación Biomédica de Málaga-IBIMA, 29010 Málaga, Spain; isabellanz@yahoo.es; 4 Centro de Investigación Biomédica en Red de Enfermedades Hepáticas y Digestivas (CIBERehd), 29010 Málaga, Spain; 5 UGC de Anatomía Patológica, Hospital Universitario Virgen de la Victoria, 29010 Málaga, Spain; 6 Servicio de Análisis Clínicos e Inmunología, UGC de Laboratorio Clínico, Hospital Universitario Virgen de las Nieves, Departamento de Bioquímica y Biología Molecular II/Inmunología, Facultad de Medicina, Universidad de Granada, 18014 Granada, Spain; fruizc@ugr.es; 7 Instituto de Investigación Biosanitaria ibs, 18014 Granada, Spain; 8 UICEC IBIMA, Plataforma SCReN (Spanish Clinical Research Network), Servicio de Farmacología Clínica, Hospital Universitario Virgen de la Victoria, Universidad de Málaga, 29010 Málaga, Spain

**Keywords:** non-alcoholic fatty liver disease (NAFLD), drug-induced liver injury (DILI), immunophenotype, immune response, liver fibrosis

## Abstract

Background: Non-alcoholic fatty liver disease (NAFLD) and idiosyncratic drug-induced liver injury (DILI) could share molecular mechanisms involving the immune system. We aimed to identify activation immunological biomarkers in invariant natural killer T (iNKT) and CD4/CD8+ T cells in NAFLD and DILI. Methods: We analyzed the activation profile (CD69, CD25, and HLA-DR) and natural killer group 2 member D (NKG2D) on iNKT cells, and CD4/CD8 T cells in peripheral blood mononuclear cells from NAFLD, with or without significant liver fibrosis, and DILI patients. Results: There was an increase in iNKT cells in NAFLD patients compared to DILI or control subjects. Regarding the cellular activation profile, NAFLD with significant liver fibrosis (F ≥ 2) displayed higher levels of CD69+iNKT cells compared to NAFLD with none or mild liver fibrosis (F ≤ 1) and control patients. CD69+iNKT positively correlated with insulin resistance, aspartate aminotransferase (AST) level, liver fibrosis-4 index (FIB4) and AST to Platelet Ratio Index (APRI). DILI patients showed an increase in CD69+ and HLA-DR+ in both CD4+ and CD8+ T cells, detecting the most relevant difference in the case of CD69+CD8+ T cells. Conclusions: CD69+iNKT may be a biomarker to assess liver fibrosis progression in NAFLD. CD69+CD8+ T cells were identified as a potential distinctive biomarker for distinguishing DILI from NAFLD.

## 1. Introduction

Non-alcoholic fatty liver disease (NAFLD), recently renamed by some authors as a metabolic-associated fatty liver disease [1], includes a wide spectrum of histological lesions ranging from simple steatosis to nonalcoholic steatohepatitis (NASH) and cirrhosis. NAFLD can be considered as the liver component of metabolic syndrome and has emerged as the leading cause of chronic liver disease in Western countries. It is expected to rank first among patients listed for liver transplantation in the next 10 years [2]. Oxidative stress, lipid peroxidation, and mitochondrial dysfunction are important factors involved in the pathogenesis of these liver diseases. In addition, the immune response also plays a pathogenic role in NAFLD. Thus, gut microbiota dysbiosis and an altered intestinal permeability in NAFLD [3] result in the secretion of gut-derived bacterial products in the portal circulation, contributing to hepatic immune response [4].

Idiosyncratic drug-induced liver injury (DILI) is defined as unpredictable liver damage caused by drugs or other non-pharmacological compounds [5,6]. The interplay of drug properties, genetic and non-genetic host, and environmental factors in susceptible individuals favor DILI development [7]. This type of DILI occurs in a small proportion of exposed patients with a wide variety of clinical, biochemical, and histological presentations that can progress to acute liver failure leading to death or liver transplantation [8].

The relationship between NAFLD and DILI is bidirectional. Thus, NAFLD can predispose to hepatotoxicity induced by certain drugs. There are agents that can induce fatty liver disease or aggravate preexisting steatosis [9,10,11,12,13]. Moreover, the differential diagnosis between metabolic or drug-induced fatty liver disease is complex due to the absence of specific diagnostic biomarkers [14].

The reciprocal relation between these entities suggests that NAFLD and DILI may share complex pathogenic mechanisms. One of the common upstream mechanisms in liver injury is the induction of hepatic oxidative stress, which ultimately leads to innate and adaptive immune responses [15,16]. The liver tends towards immune tolerance to protect itself from chronic inflammation produced by lipotoxicity and gut-derived bacterial products. Its breakdown could be favoring the development of liver disorders [17]. The regulation of hepatic immune response is extremely complex and involves different types of cells from both the innate and the adaptive immune systems [17]. In this context, natural killer T (NKT) cells are the main innate-like T cells in the liver of both mice and humans [18]. Invariant NKT (iNKT) cells are a cell sub-population known to cause inflammation in liver [19,20]. Nonetheless, the activation profile and the mechanism by which iNKT cells are involved in NAFLD progression and DILI in humans remain unclear.

Metabolites synthesized during toxin metabolism, lipotoxicity, oxidative stress, or metabolites derived from foreign antigens and bacteria may be presented on human leukocyte antigen (HLA) molecules as neoantigens, triggering activation of T-cells and subsequent immune response. Activation of T cells is accompanied by upregulation of different cell surface markers, each at a different stage of the activation process. CD69 has been described as the earliest activation marker of T lymphocytes [21]. CD69 is a co-stimulatory molecule and promotes cytotoxicity [22]. In contrast, CD25 and HLA-DR are usually considered as late and very late activation cell surface markers on leukocytes, respectively [23,24]. CD25 is one of the most studied activation markers and has a critical role in the maintenance of regulatory T cell (Treg)-mediated immunotolerance toward self-antigens [25]. HLA-DR appears at the late stages of activation on T and NK cells and is a genetic marker involved in the host immune response [26]. On the other hand, the natural killer group 2 member D (NKG2D), a strong activating receptor, is associated with stress-induced tissue damage [27] and would be crucial in repairing liver fibrosis [28].

We aimed to better understand the pathogenic relationship between drug-induced and metabolic-induced liver injury. To that end, we aimed to unravel immune biomarkers involved in the pathogenesis of these disorders, therefore identifying new tools for the differential diagnosis of DILI and NAFLD. We have focused on the study of the expression of activation markers (CD69, CD25, HLA-DR, and NKG2D) in iNKT and CD4+ and CD8+ T cells from NAFLD patients stratified by the degree of fibrosis and DILI patients.

## 2. Material and Methods

### 2.1. Patients

The study included NAFLD patients (*n* = 21), DILI patients (*n* = 8), and healthy controls (*n* = 28) recruited prospectively (Table 1). Samples were processed and frozen immediately after their collection at the Biobank of the Virgen de la Victoria University Hospital (Andalusian Public Health System Biobank, Málaga, Spain). The present study was carried out in accordance with the Code of Ethics of the World Medical Association (Declaration of Helsinki) and was approved by the Malaga Provincial Research Ethics Committee, Spain (PI-0285-2016). All the participants gave their written informed consent. All authors had access to the study data and have reviewed and approved the final manuscript.

#### 2.1.1. Cohort of Patients with DILI

DILI patients were collected from the prospective Spanish DILI Registry. In-depth details of this registry have been described elsewhere [29]. Briefly, suspected DILI cases were assessed for (i) the compatibility of the time span between drug intake and the onset of symptoms, (ii) all biochemical, histological, and imaging data to exclude alternative (liver) diseases, and (iii) the outcome of the liver injury. Specifically, no healthy control, NAFLD and DILI patients had detectable titers of autoantibodies in serum. Afterward, the CIOMS/RUCAM (Council for International Organizations of Medical Sciences/Roussel Uclaf Causality Assessment Method) scale was applied, and finally, cases were adjudicated by three DILI experts before including them in the abovementioned DILI Registry. The biochemical DILI criteria and severity of liver injury were defined according to the well-established criteria set by an international DILI expert group [6]. DILI patients had jaundice (75%) and required hospitalization (75%). The type of liver injury (R = ALT/ULN/ALP/ULN) (ULN = upper limit of normal) was hepatocellular (R ≥ 5; 50%), cholestatic (R ≤ 2; 37%) and mixed (R > 2 and R < 5; 13%). DILI severity was mild in 25% of patients, 62.5% showed a moderate injury, and 12.5% of patients underwent liver transplantation. Culprit agents responsible of DILI were ombitasvir combination (ombitasvir, dasabuvir, paritaprevir and ritonavir) (*n* = 1), clenbuterol/amoxicillin-clavulanate (*n* = 1), dietary supplement (*n* = 1), terbinafine (*n* = 1), isoniazid (*n* = 1), levofloxacin (*n* = 1), and amoxicillin-clavulanate (*n* = 2).

#### 2.1.2. Cohort of Patients with NAFLD

Patients fulfilling invasive and non-invasive criteria for the diagnosis of NAFLD were prospectively recruited from the Gastroenterology Department of the Virgen de la Victoria University Hospital, Málaga, Spain. Inclusion criteria were [30] histological diagnosis of NASH (presence of steatosis in the liver ≥5%, hepatocellular damage, hepatocyte ballooning or presence of fibrosis in the liver biopsy) (*n* = 12) or non-invasive diagnosis of NAFLD (for those patients without liver biopsy, the diagnosis was assumed by excluding other causes of liver damage, non-invasive test and the presence of steatosis in the abdominal ultrasound) (*n* = 9). Exclusion criteria were [30] alcohol intake >20 g/day (man) and >10 g/day (woman), secondary causes of NAFLD or other causes of chronic hepatic disease, consumption of potential drugs for the development of NAFLD, patients with type 1 diabetes, and severe psychiatric disorders. We divided NAFLD patients into two groups according to the liver fibrosis degree: no significant liver fibrosis (F ≤ 1) and significant fibrosis (F ≥ 2) measured by FibroScan^®^. This classification has been chosen according to a previous study, which suggested that the risk of long-term overall mortality, liver transplantation, and liver-related events was only present after liver fibrosis progression to F2 [31].

#### 2.1.3. Cohort of Healthy Controls

Healthy controls were recruited among workers of the Virgen de la Victoria University Hospital and the University of Málaga (Málaga, Spain). Inclusion criteria were the absence of a history of liver disease. Exclusion criteria were previous history of hepatotoxicity or any other chronic liver disease, altered liver profile at the time of inclusion, body mass index >25 kg/m^2^, diabetes mellitus, dyslipidemia, metabolic syndrome, or NAFLD.

### 2.2. FibroScan Examination

Participants were examined in a fasting state. Transient elastography (TE) was performed using ECHOSENS FibroScan 402 (Echosens, Paris, France) with an M or XL probe on the right lobe of the liver. Liver stiffness measurement (LSM) were described by the median of 10 successful measurements. LSM is considered reliable only if IQR/med < 30% and success rate > 60%. Ten successful acquisitions were performed for all patients. NAFLD patients were included in two groups depending on the fibrosis level. No significant liver fibrosis (F ≤ 1) and significant liver fibrosis (F ≥ 2) based on TE defined as LSM ≥ 7 kPa, ≥8.7 kPa and ≥10.3 kPa (≥F2, ≥F3 and F4, respectively) for M probe, and LSM ≥ 6.2 kPa, ≥7.2 kPa and ≥7.9 kPa (≥F2, ≥F3 and F4, respectively) for XL probe [32].

### 2.3. Steatosis, NASH, and Fibrosis Non-Invasive Tests

The fatty liver index (FLI) was calculated to assess hepatic steatosis [33]. To evaluate liver fibrosis, the NAFLD fibrosis score (NAFLD FS) [34], the fibrosis-4 index (FIB4) [35], and the AST-to-platelet ratio index (APRI) [36] were used.

### 2.4. Biochemical Measurements

Blood samples from all patients were collected in a fasting state. In DILI patients, blood samples were collected between days 1–11 from DILI recognition. Blood was collected in sodium heparin and serum tubes to isolate cellular fraction and serum, respectively. The serum was isolated and frozen at −80 °C. Biochemical variables were measured in duplicate in a modular analytics E170 analyzer (Roche Diagnostics GmbH, Mannheim, Germany). HOMA-IR was calculated using the following equation: HOMA-IR = fasting insulin (µIU/mL) × fasting glucose (mmol/L)/22.5.

### 2.5. Cellular Fraction Isolation and Flow Cytometry Study

Peripheral blood mononuclear cells (PBMCs) were isolated from total blood collected in sodium heparin tubes by density gradient (Lymphoprep™, STEMCELL Technologies Inc., Vancouver, BC, Canada). Once isolated, cells (2 × 10^6^) were resuspended in PBS 1X and incubated with anti- CD45-V450, CD3-APC-Cy™7, CD4-PerCP-Cy™5.5, CD25-PE-Cy™7, CD69-APC, NKG2D-PE-Cy™7, HLA-DR-APC (BD Biosciences, San Jose, CA, USA), TCRVβ11-FITC, and TCRVα24-PE (Beckman-Coulter, Marseille, France). Gating strategies for primary antibodies calibration by flow cytometry show in Figure S1. The following isotype controls were included: IgG1-V450, IgG1-APC-Cy™7, IgG1-PerCP-Cy™5.5, IgG1-PE-Cy™7, IgG1-APC, IgG2-APC (BD Biosciences, San Jose, CA, USA), IgG1-PE, IgG2a-FITC (Beckman-Coulter, Marseille, France). Samples were incubated at 1:100 dilutions with each antibody for 45 min–1 h at room temperature. One million events were acquired. Cells were gated based on the expression of specific markers. CD3 cell subpopulations were designated by the selection of CD45+CD3+ cells in the lymphocyte gate (Figure S2). iNKT cells were recognized as CD3+TCRV-α24+TCRV-β11+ cells (Figure S2) [37]. Expression of early (CD69+) and late (CD25 and HLA-DR) activation cell surface markers and cellular stress marker (NKG2D) was carried out in iNKT cells, CD4+ and CD8+ T cells subsets. Gating strategies are described in Figures S2 and S3. Data were analyzed using KALUZA 2.1 Software (Beckman-Coulter, Indianapolis, IN, USA). Results were shown as the percentage of positive cells to each marker among each subset collected.

### 2.6. Statistical Analysis

Data were analyzed with GraphPad Prism 7.04 (GraphPad Software, San Diego, CA, USA) and R version 4.1.1 (R Core Team, Vienna, Austria, 2021). Differences between groups were compared using Kruskal-Wallis tests followed by post hoc analyses using the Dunn’s test. The Bonferroni correction for multiple comparisons was applied. Spearman correlation coefficients were calculated to estimate the relationship between variables. A *p*-value < 0.05 was deemed as statistically significant.

## 3. Results

### 3.1. iNKT Cells Are Increased in NAFLD Patients with Significant Fibrosis

**Figure 1:**
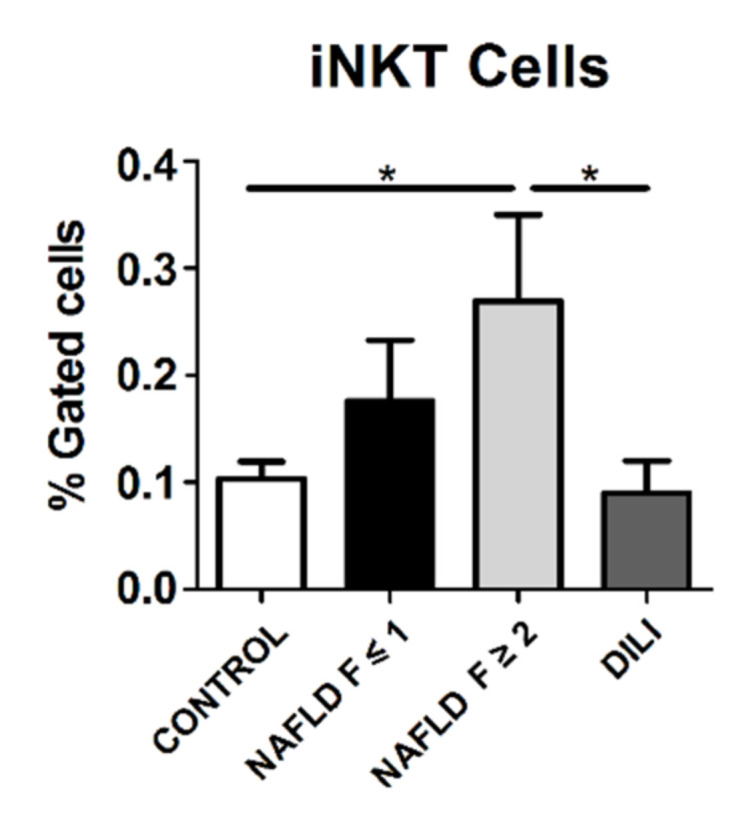
Percentage of invariant natural killer T (iNKT) cells in T lymphocytes from peripheral blood mononuclear cells from healthy controls, non-alcoholic fatty liver disease (NAFLD) and drug-induced liver injury (DILI) patients. Data are presented as mean ± standard error of the mean. * *p* ≤ 0.05.

First, we analyzed the percentage of iNKT cells among lymphocytes from PBMCs. The frequency of iNKT cells was significantly higher in NAFLD patients with significant liver fibrosis (F ≥ 2) (mean % ± standard deviation [SD]; 0.27 ± 0.08) with regard to DILI patients (0.09 ± 0.03, *p* = 0.035) and healthy controls (0.11 ± 0.02, *p* = 0.031) (Figure 1).

### 3.2. iNKT Cells from NAFLD Patients with Significant Fibrosis Presented Increased Early Activation Profile

**Figure 2:**
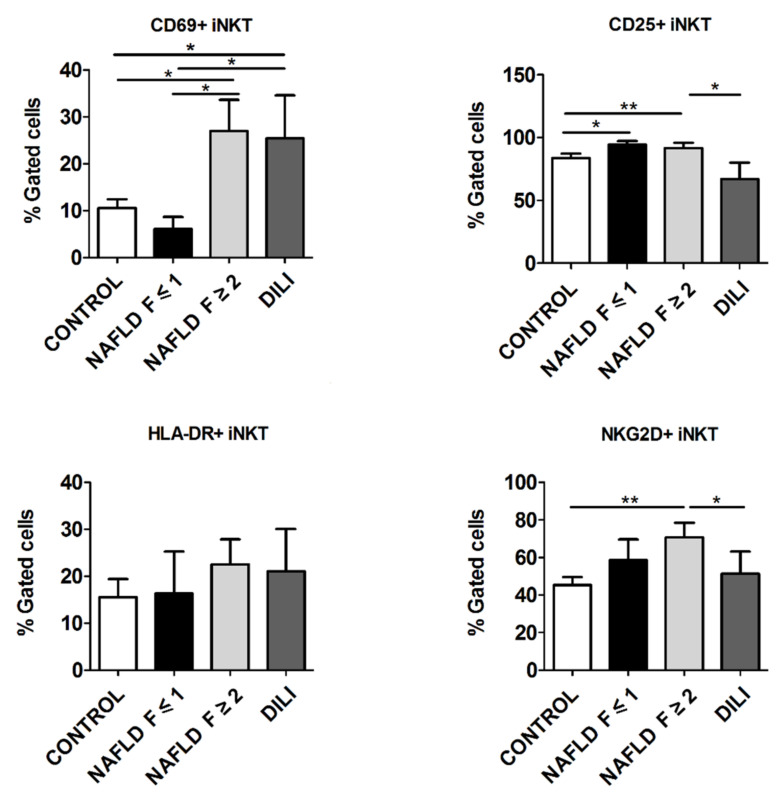
Expression of early (CD69+) and late (CD25+, HLA-DR+) activation markers, and NKG2D+ in invariant natural killer T (iNKT) cells in T lymphocytes from peripheral blood mononuclear cells from healthy controls, non-alcoholic fatty liver disease (NAFLD) and drug-induced liver injury (DILI) patients. Data are presented as mean ± standard error of the mean. * *p* ≤ 0.05 and ** *p* ≤ 0.001.

Interestingly, NAFLD patients with significant liver fibrosis (F ≥ 2) had increased the percentage of CD69+iNKT cells (26.9 ± 6.6) with respect to those without significant liver fibrosis (F ≤ 1) (6.1 ± 2.5, *p* = 0.040) and healthy control (10.1 ± 1.9, *p* = 0.031) (Figure 2). There was also an increase in the percentage of CD69+iNKT cells in DILI patients (25.3 ± 9.4) with respect to NAFLD patients without significant liver fibrosis (F ≤ 1) (6.1 ± 2.5, *p* = 0.045) and healthy controls (10.1 ± 1.9, *p* = 0.042) (Figure 2).

### 3.3. iNKT Cells from NAFLD Patients Presented Increased Late Activation Profile

The frequency of CD25+iNKT cells was increased in NAFLD patients, regardless of the degree of fibrosis, compared to healthy controls (for F ≤ 1 group: 94.3 ± 2.9 vs. 83.2 ± 3.5, *p* = 0.043; and for F ≥ 2 group: 91.5 ± 4.3 vs. 83.2 ± 3.5, *p* = 0.001) (Figure 2). Also, NAFLD patients with significant liver fibrosis (F ≥ 2) presented a marked increase in the percentage of CD25+iNKT compared to DILI patients (91.5 ± 4.3 vs. 66.8 ± 13.1, *p* = 0.046) (Figure 2). No significant differences in the percentage of HLA-DR+iNKT cells between NAFLD and DILI patients or healthy controls were found (Figure 2).

### 3.4. iNKT Cells from NAFLD Patients Presented Increased Cellular Stress

As shown in Figure 2, the percentage of NKG2D+iNKT cells was increased in NAFLD patients with significant liver fibrosis (F ≥ 2) (70.6 ± 7.7) as compared to DILI patients (50.9 ± 12.1, *p* = 0.016) and healthy controls (44.6 ± 4.2, *p* = 0.001).

### 3.5. CD69+ and HLA-DR+ CD4+ T Cells Subset Is Increased in DILI Patients

**Figure 3:**
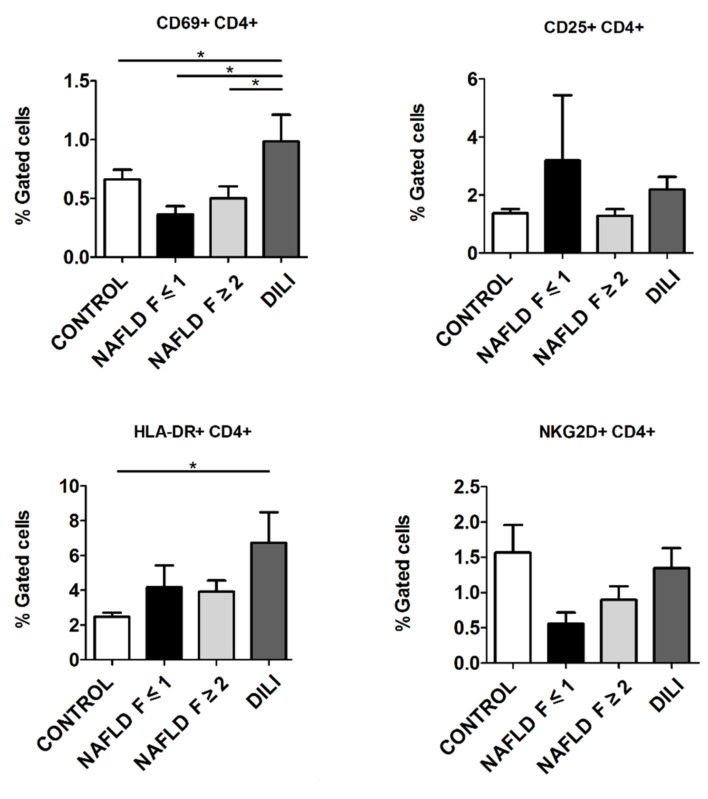
Expression of early (CD69+) and late (CD25+, HLA-DR+) activation markers, and NKG2D+ in CD4+ T cells in T lymphocytes from peripheral blood mononuclear cells from healthy controls, non-alcoholic fatty liver disease (NAFLD) and drug-induced liver injury (DILI) patients. Data are presented as mean ± standard error of the mean. * *p* ≤ 0.05.

We next sought to assess the activation profile of CD4+ T cells in NAFLD and DILI patients. There was a significant increase in the percentage of CD69+CD4+ T cells in DILI patients compared to NAFLD patients (for F ≤ 1 group: 0.97 ± 0.27 vs. 0.36 ± 0.08, *p* = 0.011; and for F ≥ 2 group: 0.97 ± 0.27 vs. 0.50 ± 0.10 *p* = 0.007) and healthy controls (0.97 ± 0.27 vs. 0.67 ± 0.11, *p* = 0.045) (Figure 3).

We found a significant increase in the percentage of HLA-DR+CD4+ T cells in DILI patients compared to healthy controls (6.7 ± 1.8 vs. 2.5 ± 0.2, *p* = 0.002) (Figure 3).

There were no significant changes in the expression of late activation marker CD25 or the cellular stress marker NKG2D in CD4+ T cells across the different groups (Figure 3).

### 3.6. CD69+ and HLA-DR+ CD8+ T Cells Subset Is Increased in NAFLD and DILI Patients

**Figure 4:**
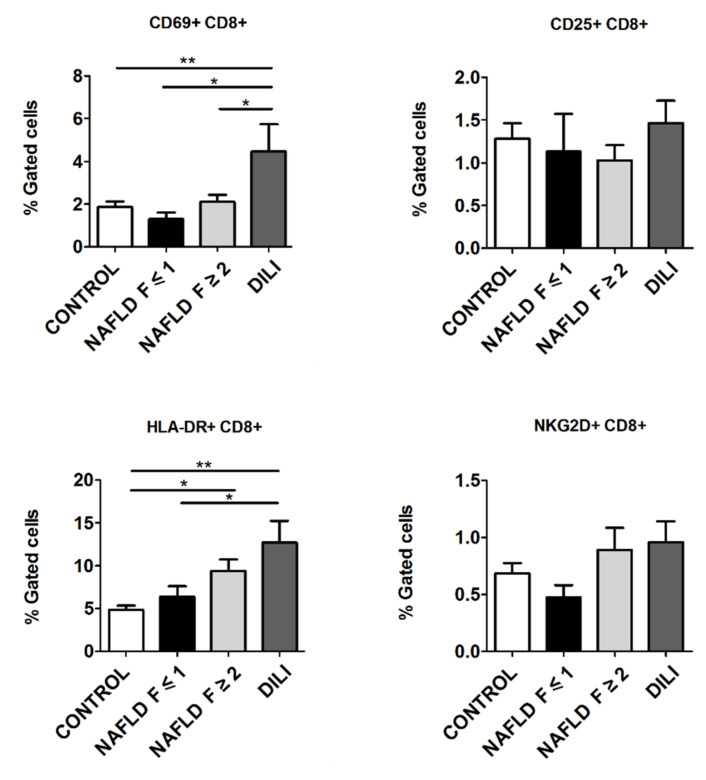
Expression of early (CD69+) and late (CD25+, HLA-DR+) activation markers, and NKG2D+ in CD8+ T cells in T lymphocytes from peripheral blood mononuclear cells from healthy controls, non-alcoholic fatty liver disease (NAFLD) and drug-induced liver injury (DILI) patients. Data are presented as mean ± standard error of the mean. * *p* ≤ 0.05, ** *p* ≤ 0.001.

CD8+ T cells showed an activation profile similar to that observed in CD4+ T cells. CD69+CD8+ T cells were notably increased in DILI patients compared to NAFLD patients (for F ≤ 1 group: 4.5 ± 1.3 vs. 1.3 ± 0.3, *p* = 0.006; and for F ≥ 2 group: 4.5 ± 1.3 vs. 2.1 ± 0.3, *p* = 0.032) and healthy controls (4.5 ± 1.3. vs. 1.9 ± 0.3, *p* = 0.001) (Figure 4).

In addition, HLA-DR+CD8+ T cells were significantly increased in DILI patients (12.6 ± 2.6) compared to NAFLD patients without significant liver fibrosis (F ≤ 1) (6.9 ± 1.1, *p* = 0.045) and healthy controls (5.0 ± 0.5, *p* < 0.001) (Figure 4). Besides, a significant increase was found in NAFLD patients with significant liver fibrosis (F ≥ 2) with regard to healthy controls (9.0 ± 1.3 vs. 5.0 ± 0.5, *p* = 0.006) (Figure 4). However, no significant differences were found in the percentage of CD25+CD8+ and NKG2D+CD8+ T cells among groups (Figure 4).

### 3.7. Correlations between the Activation Profile of Lymphocytes with Anthropometric/Biochemical Variables and Fatty Liver and Fibrosis Index

**Figure 5:**
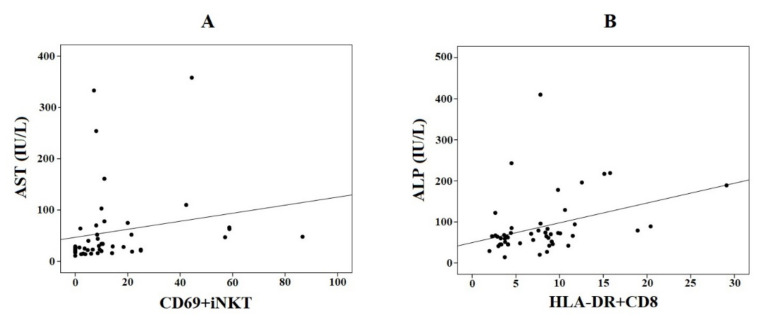
Spearman’s correlations between CD69+iNKT and aspartate aminotransferase (AST) (**A**) and between HLA-DR+CD8+ and alkaline phosphatase (ALP) (**B**) for the whole population (non-alcoholic fatty liver disease (NAFLD), drug-induced liver injury (DILI), and healthy controls).

**Figure 6:**
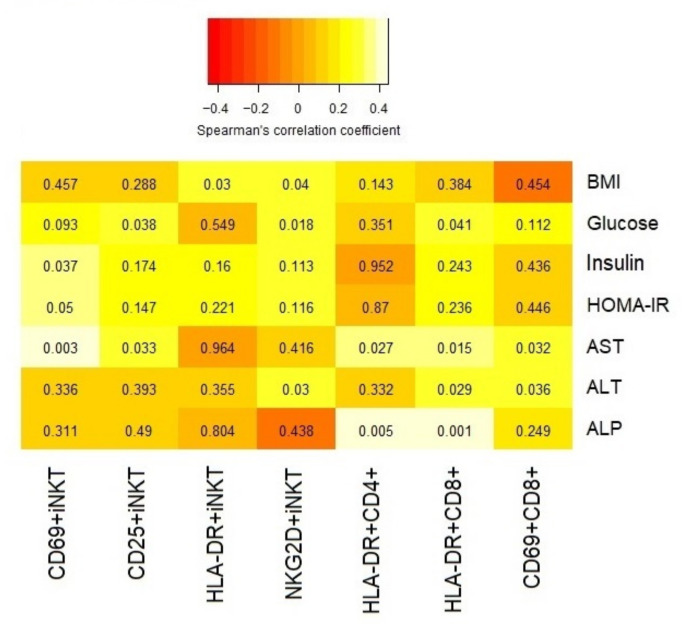
Spearman’s correlations between the activation profiles of iNKT cells, CD4+ and CD8+ T lymphocytes, and anthropometric/biochemical variables for the whole population (non-alcoholic fatty liver disease (NAFLD), drug-induced liver injury (DILI), and healthy controls) (*p*-value in each cell).

Positive moderate correlations (r > 0.4) were found between CD69+iNKT and AST (r = 0.43, *p* = 0.003) (Figure 5A), and between HLA-DR+CD8+ and ALP (r = 0.45, *p* = 0.001) (Figure 5B). Notably, there were marginal low-to-moderate correlations (0.35 < r < 0.4) between CD69+iNKT and either insulin (r = 0.37, *p* = 0.037) and HOMA-IR (r = 0.35, *p* = 0.049), between HLA-DR+CD4+ and ALP (r = 0.39, *p* = 0.005), and between HLA-DR+CD8+ and AST (r = 0.36, *p* = 0.015). The remaining correlations are shown in Figure 6.

When only NAFLD patients were considered, the positive moderate correlation between CD69+iNKT and AST persisted although not significant (r = 0.45, *p* = 0.068). Conversely, in this subgroup of patients, ALT levels were inversely correlated with the expression of both HLA-DR+CD8+ (r = −0.68, *p* = 0.001) and HLA-DR+CD4+ (r = −0.47, *p* = 0.031).

To analyze the correlation with fatty liver and fibrosis index, we only considered controls and NAFLD patients, and excluded the group with high level of liver enzymes (DILI group). Thus, when excluding DILI patients, activation of CD25+iNKT was positively correlated with higher scores of FLI (r = 0.43; *p* = 0.021) and APRI (r = 0.38; *p* = 0.021), as well as between CD69+iNKT and FIB4 index (r = 0.39; *p* = 0.018) and APRI (r = 0.37, *p* = 0.025).

## 4. Discussion

The interplay between metabolic syndrome, NAFLD, and DILI is complex, and it is still unclear whether NAFLD is a risk factor for DILI development [9,10,11,12,13,38]. The important role that the immune system plays in both, NAFLD and DILI, as well as the similar clinical steatosis profile that certain drugs induce in the liver, suggest that they may share molecular mechanisms involving the immune system. Therefore, it is critical to characterize the role of the immune system in both diseases to better distinguish DILI cases from those with primary NAFLD.

Maricic et al. suggested the involvement of the immune response mediated by iNKT cells in the progression from steatosis to non-alcoholic steatohepatitis (NASH) and fibrosis in an experimental murine model and human NAFLD patients [19]. iNKT cells are pro-inflammatory and can promote liver injury [39]. In agreement with these previous results, we have found increased levels of iNKT cells in PBMC from NAFLD patients with significant liver fibrosis, suggesting a role of iNKTs in the evolution of NAFLD. However, our study fails to detail the molecular pathways involved in the increase of iNKT cells in peripheral blood. This increase could be due to the loss of gut integrity and heavy exposure to bacterial antigens and metabolites, as well as to clean free lipids in the proinflammatory microenvironment observed during steatohepatitis [40,41]. On the other hand, DILI patients showed a similar percentage of iNKT cells than control subjects, but lower than NAFLD patients with higher fibrosis. In regard to DILI, there are conflicting results. While some studies show an increase in iNKT after triptolide [42] and alpha-naphthylisothiocyanate treatment [20], others observe a decrease in acetaminophen-treated mice [43]. However, these studies were carried out in the liver of animal models, and data on peripheral iNKT cells were not reported. Our study, conducted in human PBMCs, suggests a more relevant role of iNKT cells in the pathogenesis of NAFLD compared to DILI, which may represent a differential immunological feature between these two entities.

Activation of iNKT cells is a crucial mechanism in the progression from steatosis to steatohepatitis and fibrosis, both in peripheral blood and in the liver [19]. Although our peripheral blood data could be a reflection of what is happening in the liver, we have no immunohistochemical data to support this hypothesis. However, the correlations found with liver parameters would point in this direction. Upon activation, iNKT cells can exhibit a potent pro-inflammatory effector function [44]. Previous studies have shown that the frequency of T cells (CD3+) expressing CD69 was higher in the liver of methionine-choline deficient-fed mice, a widely-used mouse model of NASH [45]. Our results also found a higher expression of CD69 in peripheral iNKT cells from NAFLD patients with significant liver fibrosis. Furthermore, we found a positive correlation between CD69 expression in iNKT cells and FIB4, a liver fibrosis index, and AST, a strong predictor of hepatocellular injury and acute liver failure [46,47]. These correlations reinforce our hypothesis of the possible role of CD69+iNKT as a biomarker of liver fibrosis progression. 

The nature of the antigens that would stimulate iNKT cells is complex. In fact, mouse and human iNKT cells can recognize lipid and glycolipid antigens of self or microbial origin presented on MHC class-I-like CD1d molecules [48,49]. In this sense, intestinal permeability appears to be increased in NAFLD patients, which would allow a constant exposure to dietary and microbial antigens. This altered permeability is associated with the degree of hepatic steatosis [50] and could be responsible for the increased CD69+ activation of iNKT cells. However, iNKT cells are also located in the liver, where lipid metabolism is active, and in adipose tissue, another location for lipid metabolism with endocrine functions. Our data would support the fact that iNKT cells play an important role in a disease that involves abnormal lipid metabolism or lipid-related inflammation, as is the case of NAFLD, and to a much lesser extent in DILI, in which other cell types of the classical adaptive response would preferentially participate, such as CD8 and CD4 T cells. However, our study was not designed to draw such a conclusion and this hypothesis needs to be assessed in future functional studies. 

The positive correlations found between CD69+iNKT and either insulin and HOMA-IR reinforce the role of this cell type in the evolution of NAFLD. It is known that elevated insulin and insulin resistance are involved in the metabolic etiology of NAFLD. In states of insulin resistance, one of the main events that occur in the liver is an excessive accumulation of free fatty acids and further conversion to toxic metabolites. This can trigger oxidizing, inflammatory, fibrotic, and apoptotic signaling pathways, which would lead to a progression from NAFLD to NASH [13].

In this context of stress-induced progression to fibrosis, NKG2D has a relevant role in ameliorating liver fibrosis [28]. NKG2D is a marker of stress-induced tissue damage [27]. We have observed a marked increase in NKG2D+iNKT levels in NAFLD patients with higher liver fibrosis, which parallels the results found by Stiglund et al. [51]. They showed an upregulation of NKG2D in NK cells from peripheral blood in NASH patients [51], but not in patients with fatty liver, in which NKG2D levels were similar to those found in healthy controls. They postulated that increased NKG2D expression could be a reaction to the enlarged hepatocyte stress, inflammation, and gut microbiota-derived signals observed in NASH patients [52]. The positive correlations between NKG2D+iNKT and serum glucose and BMI, which are also surrogate markers of stress and chronic inflammation [53,54], could suggest the activation of NKG2D in response to liver fibrosis.

In the DILI group, the most striking result was the increase in CD69+ and HLA-DR+ in both CD4+ and CD8+ T cells, which may be used as a biomarker to differentiate between NAFLD and DILI patients, particularly in those presenting mild-to-moderate elevations in transaminases. HLA molecules have a relevant role in the susceptibility to DILI and may explain the different patterns of liver damage at presentation [26,55,56]. Interestingly, we found a significant upregulation of HLA-DR in CD4+ and CD8+ T cells in DILI, underscoring the role of the adaptive immune response in DILI [57].

We could hypothesize that the increase in CD69+CD4+ and HLA-DR+CD4+ could be explained by the appearance of neoantigens after the interaction of the drug with proteins, as evidenced by its association with HLA, at an early stage of drug exposure. It would therefore be an antigenic stimulus that may be before liver damage has yet occurred. Fibrosis is never a process that is detected in the initial stages and would reflect more the result of chronic and persistent stimulation, probably tending to a change in the polarization of both macrophages and iNKT cells towards a Th2 profile, which is related to the fibrosis. However, the fact that there is an increase in the CD8 population, and to a lesser extent the CD4 population, suggests rather the participation of Th1 cytokines. Despite CD4+ cells being necessary for an immune response, CD8+ cytotoxic T cells are considered the major effector cells [58]. Neoantigens synthesized in hepatocytes by reactive metabolites are also presented by HLA class-I to CD8, which is consistent with the characteristic histology of DILI, rich in CD8+ T cells [59]. In addition, many of the HLA associations that predict the risk of DILI suggest that most of the liver injury in DILI is caused by a CD8+ cell-mediated immune response [58]. Moreover, the depletion of CD8+ T cells protected mice from induced liver injury, which strongly suggests that CD8+ T cells are partly responsible for liver damage [59]. Our results fit in this context with the increase in CD69+CD8+ T cells, suggesting that their activation profile is involved in DILI.

A limitation of our study is the relatively small size of the included cohorts. Despite DILI is a rare event, the patients enrolled in the study constitute a well-vetted population with a long-term follow-up. Indeed, despite the limited sample size, the results were consistent and did not show significant deviations, allowing after multiple testing corrections to obtain solid results. In addition, the iNKT cell subset and the activation markers evaluated have been poorly investigated in human populations. In addition, it would be interesting for further studies to be able to include a control group with characteristics similar to those of the NALFD group (higher age and BMI), but without insulin resistance or liver abnormalities.

## 5. Conclusions

This is the first study showing a comparative immunophenotypic profile of the activation of iNKT cells, CD4+ and CD8+ T cells in PBMC from NAFLD with different degrees of liver fibrosis and DILI patients. We found an increase in iNKT and CD69+iNKT cells in NAFLD patients with significant liver fibrosis, suggesting that CD69+iNKT cells could be a biomarker of liver fibrosis progression in NAFLD. Our findings identified CD69+CD8+ T cells as a potential new biomarker, which could improve the non-invasive diagnosis of hepatotoxicity. This study shows clear involvement of immune mechanisms for these liver diseases. Future studies including NAFLD patients with superimposed DILI to unravel mechanistic differences between DILI patients with or without prior NAFLD would be clearly warranted.

## Figures and Tables

**Table 1:** Anthropometric and biochemical variables across the different study groups.

	Healthy Control	NAFLD F ≤ 1	NAFLD F ≥ 2	DILI	*p*-Value
N (men/women)	28 (11/17)	6 (3/3)	15 (7/8)	8 (1/7)	<0.001
Age (years)	40 ± 11	48 ± 11 ^a^	56 ± 11 ^a^	47 ± 15 ^a,c^	<0.001
Weight (kg)	67 ± 12	79 ± 14 ^a^	84 ± 10 ^a^	68 ± 12 ^b,c^	<0.001
BMI (kg/m^2^)	23 ± 2	28 ± 4 ^a^	31 ± 3 ^a^	24 ± 3 ^b,c^	<0.001
Glucose (mg/dL)	82 (76–88)	98 (89–107) ^a^	106 (91–121) ^a^	94 (89–99) ^a,c^	<0.001
Cholesterol (mg/dL)	166 ± 36	197 ± 28 ^a^	191 ± 40 ^a^	184 ± 67	<0.047
Triglycerides (mg/dL)	85 ± 31	118 ± 59 ^a^	156 ± 87 ^a^	186 ± 100 ^a^	<0.001
Insulin (μU/mL)	5.7 (4.1–7.3)	11.3 (6.3–16.3) ^a^	17.8 (8.8–26.8) ^a^	11.4 (2.4–20.4)	<0.001
HOMA-IR	1.1 (0.7–0.5)	2.9 (2.7–4.1) ^a^	6.7 (4.2–9.3) ^a,b^	4.4 (0.9–7.9)	<0.001
AST (IU/L)	20 (16–24)	33 (24–42) ^a^	48 (29–67) ^a,b^	96 (41–151) ^a,b,c^	<0.001
ALT (IU/L)	22 (14–30)	58 (20–96) ^a^	74 (44–104) ^a^	202 (52–352) ^a,b,c^	<0.001
GGT (IU/L)	13 (9–17)	44 (5–83) ^a^	96 (56–136) ^a^	105 (15–195) ^a^	<0.001
ALP (IU/L)	48 (36–60)	74 (63–85) ^a^	75 (55–95) ^a^	178 (103–253) ^a,b,c^	<0.001
Hepatic Parameters Fibrosis					
FLI	18 ± 20	64 ± 28 ^a^	83 ± 19 ^a,b^	Nc	<0.001
NAFLD FS	−3.1 ± 0.7	−2.0 ± 0.8 ^a^	−1.1 ± 1.4 ^a,b^	Nc	0.001
FIB4	0.67 (0.45–0.89)	0.94 (0.65–0.1.23)	1.42 (1.02–1.82) ^a^	Nc	<0.001
APRI	0.24 (0.17–0.31)	0.43 (0.28–0.58) ^a^	0.64 (0.5–0.78) ^a^	Nc	<0.001

Results are expressed as mean ± SD or median and interquartile range. ^a^ *p* < 0.05: significant differences with respect to healthy control group. ^b^ *p* < 0.05: significant differences with respect NAFLD F ≤ 1 group. ^c^ *p* < 0.05: significant differences with respect NAFLD F ≥ 2 group. NAFLD: non-alcoholic fatty liver disease, BMI: body mass index, DILI: drug-induced liver injury, HOMA-IR: homeostasis model assessment of insulin resistance. AST: aspartate aminotransferase, ALT: alanine aminotransferase, GGT: gamma-glutamyltransferase, ALP: alkaline phosphatase; FLI: fatty liver index, NAFLD FS: NAFLD fibrosis score, FIB4: fibrosis-4, APRI: AST-to-platelet ratio index. Nc: Not calculated.

## Data Availability

The data presented in this study are available on request from the corresponding author. The data are not publicly available due to ethical reasons.

## Supplementary Materials

The following are available online at https://www.mdpi.com/article/10.3390/biomedicines10010055/s1. Figure S1: Gating strategies for primary antibodies calibration for flow cytometry, Figure S2: Gating strategies for the identification of iNKT cells and the assessment of activation marker by flow cy-tometry, Figure S3: Gating strategies for CD4+ and CD8+T cells identification among lymphocytes from peripheral blood mononuclear cells and the study of activation markers.
