# Supplementary material for: Differential iNKT and T Cells Activation in Non-Alcoholic Fatty Liver Disease and Drug-Induced Liver Injury
Source: Biomedicines. 2021 Dec 28;10(1):55. doi: 10.3390/biomedicines10010055 (PMC8772872; doi:10.3390/biomedicines10010055)
Supplement: Supplementary file 1 [file biomedicines-10-00055-s001.zip › biomedicines-1513941-supplementary.pdf]

Supplementary Materials

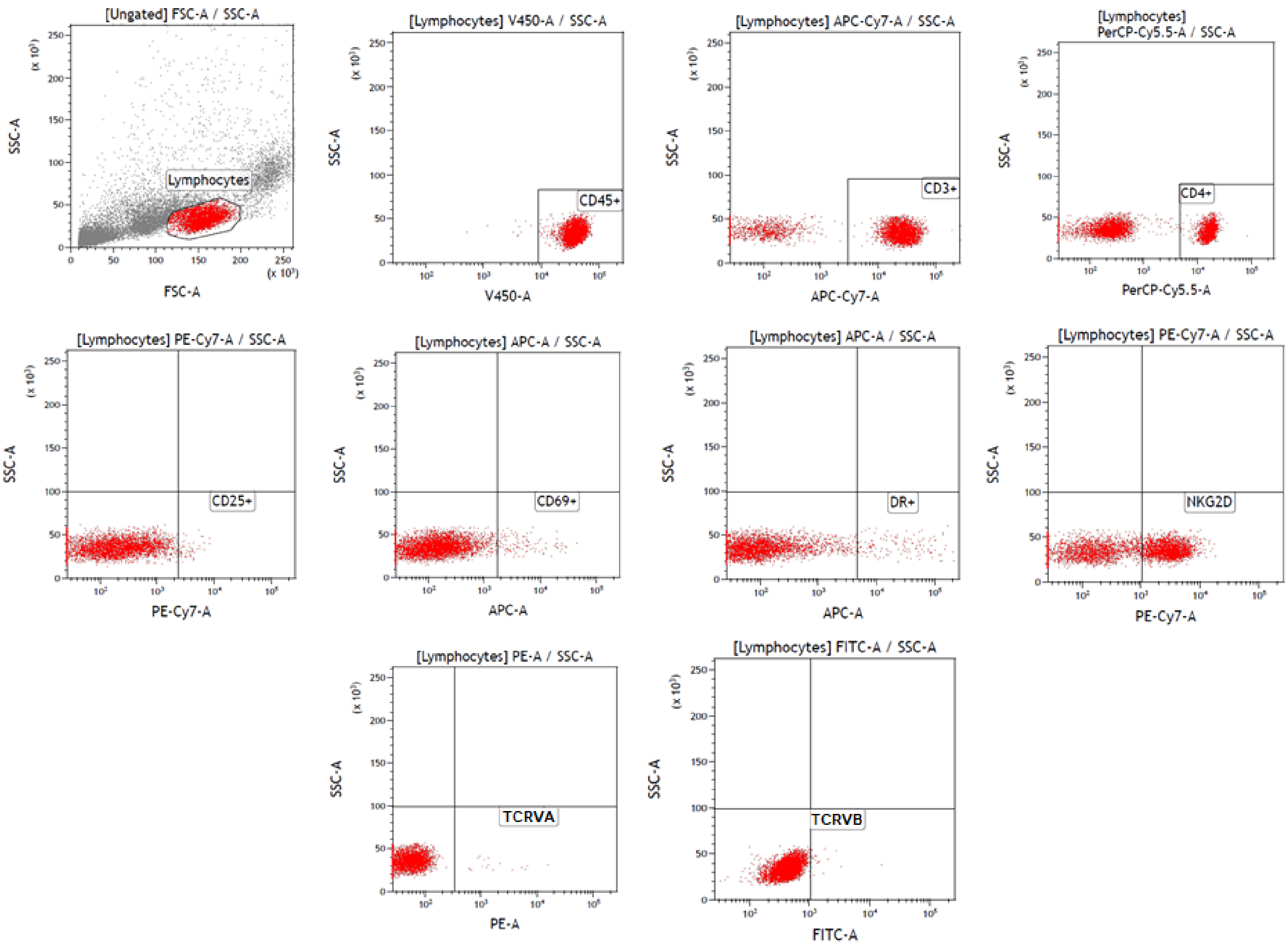

Figure S1. Gating strategies for primary antibodies calibration by flow cytometry. To test correct antibody dilution and positive cells stained, we first incubated sample with each antibody. All antibodies were incubated at 1:100 during at least 45 min. TCRVα24 and TCRVβ11 were incubated at 1:75. Representative dot plot panels show fluorescence intensity detected at each antibody. This assessment allowed us to separate negative and positive signal in order to gate specific cells subsets. Lymphocyte gate was defined based on forward (FSC) versus side scatter (SSC) complexity. Second panel shows CD45+ cells among lymphocytes from peripheral blood mononuclear cells. The rest panel show CD3+, CD4+, CD25+, CD69+, HLA-DR+, NKG2D+, TCRVα24+ and TCRVβ11+ cells among lymphocytes.

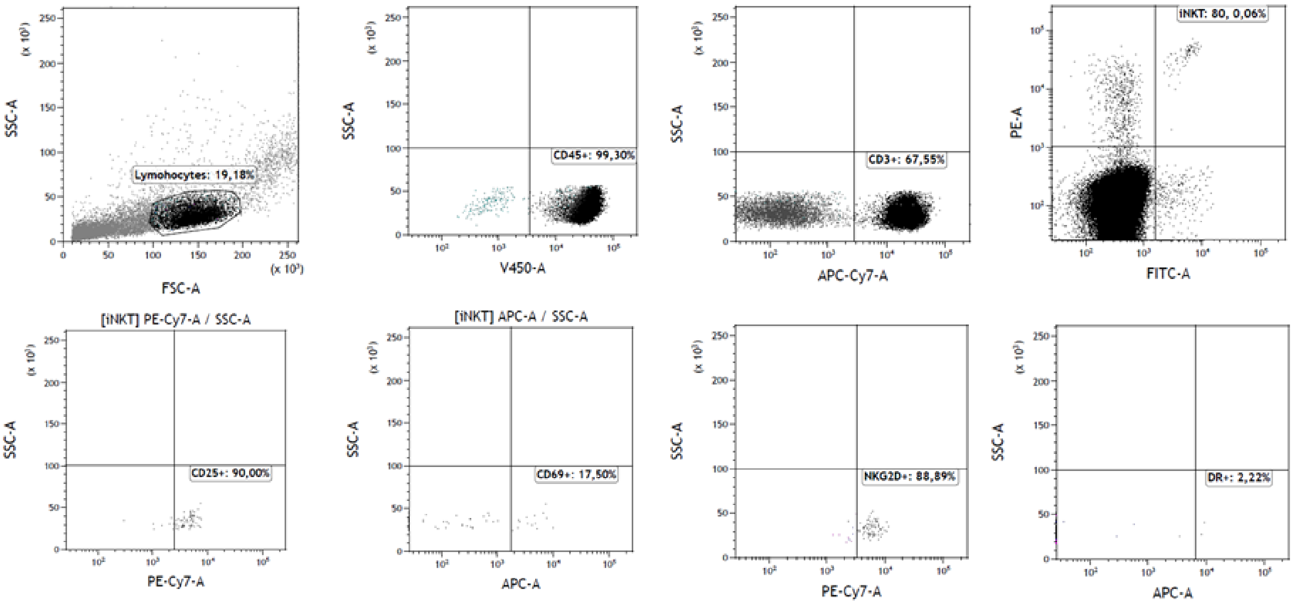

Figure S2. Gating strategies for the identification of iNKT cells and the assessment of activation markers by flow cytometry. All panels are representative of gating strategies. First, a lymphocyte gate was defined based on forward (FSC) versus side scatter (SSC) complexity. A minumum of 1 million events/sample were collected. Second, CD45+ cells among lymphocytes were selected. Third, we identified CD3+CD45+ cells among lymphocytes. Fourth, in this sub-population, we collected TCRVα24+TCRVβ11+ cells to define iNKT cells subset. Then, we analyzed the expression of CD25+, CD69+, HLA-DR+ and NKG2D+ in order to study the activation profile of iNKT cells subset.

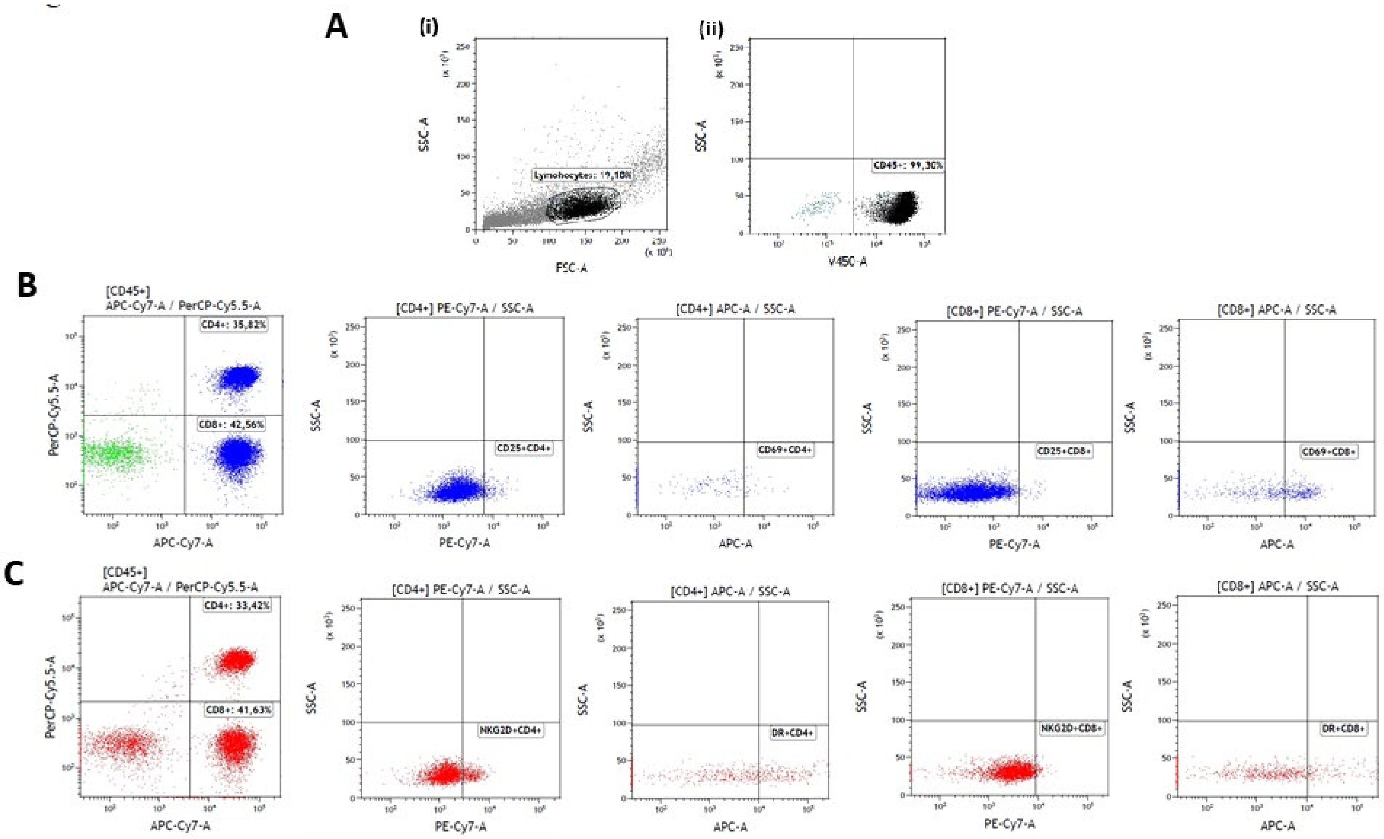

Figure S3. Gating strategies for CD4+ and CD8+ T cells identification among lymphocytes from peripheral blood mononuclear cells and the study of activation markers. Representative gating strategy. (**A**) We identified lymphocytes and CD45+ cells as we have described in Figure S1. (**i**) Lymphocytes were identified through forward versus side scatter (FSC vs. SSC) gating based on size and granularity (complexity). (**ii**) Herein, we used CD45+ to identify leukocyte populations.

Then we selected CD4+CD3+ T cells subset among CD45+ positive cells as we show in panel A, and identified CD8+ T cells as CD3+CD4− T cells. (**B**) In CD4+CD3+ and CD8+CD3+ cells we identified activation markers CD25+ and CD69+ (**B**), and NKG2D+ and HLA-DR+ (**C**).
